# Antibodies Recognizing *Mycobacterium avium paratuberculosis* Epitopes Cross-React with the Beta-Cell Antigen ZnT8 in Sardinian Type 1 Diabetic Patients

**DOI:** 10.1371/journal.pone.0026931

**Published:** 2011-10-27

**Authors:** Speranza Masala, Daniela Paccagnini, Davide Cossu, Vedran Brezar, Adolfo Pacifico, Niyaz Ahmed, Roberto Mallone, Leonardo A. Sechi

**Affiliations:** 1 Sezione di Microbiologia e Virologia, Dipartimento di Scienze Biomediche, Università degli Studi di Sassari, Sassari, Italy; 2 INSERM, U986, DeAR Lab Avenir, Cochin/Saint Vincent de Paul Hospital, Paris, France; 3 Université Paris Descartes, Sorbonne Paris Cité, Paris, France; 4 Servizio di Diabetologia, Istituto di Clinica Medica, Università degli Studi di Sassari, Sassari, Italy; 5 School of Life Sciences, University of Hyderabad, Gachibowli, Hyderabad, India; 6 Assistance Publique - Hôpitaux de Paris, Hôtel Dieu Hospital, Department of Diabetology, Paris, France; Institut national de la santé et de la recherche médicale (INSERM), France

## Abstract

The environmental factors at play in the pathogenesis of type 1 diabetes (T1D) remain enigmatic. *Mycobacterium avium* subspecies *paratuberculosis* (MAP) is transmitted from dairy herds to humans through food contamination. MAP causes an asymptomatic infection that is highly prevalent in Sardinian T1D patients compared with type 2 diabetes (T2D) and healthy controls. Moreover, MAP elicits humoral responses against several mycobacterial proteins. We asked whether antibodies (Abs) against one of these proteins, namely MAP3865c, which displays a sequence homology with the β-cell protein zinc transporter 8 (ZnT8) could be cross-reactive with ZnT8 epitopes. To this end, Ab responses against MAP3865c were analyzed in Sardinian T1D, T2D and healthy subjects using an enzymatic immunoassay. Abs against MAP3865c recognized two immunodominant transmembrane epitopes in 52–65% of T1D patients, but only in 5–7% of T2D and 3–5% of healthy controls. There was a linear correlation between titers of anti-MAP3865c and anti-ZnT8 Abs targeting these two homologous epitopes, and pre-incubation of sera with ZnT8 epitope peptides blocked binding to the corresponding MAP3865c peptides. These results demonstrate that Abs recognizing MAP3865c epitopes cross-react with ZnT8, possibly underlying a molecular mimicry mechanism, which may precipitate T1D in MAP-infected individuals.

## Introduction

Type 1 diabetes (T1D) is a paradigmatic example of autoimmune disease stemming from a complex interaction between genetic and environmental factors [1]. While several genetic susceptibility loci have been pinpointed, the environmental factors at play remain boldly elusive. Yet, environmental factors play a prominent role in T1D pathogenesis, as suggested by the incomplete (∼65%) T1D concordance between monozygotic twins [2], by migrant studies [3] or by the decreasing weight of susceptible and protective HLA Class II haplotypes over the last decades [4].

Among the environmental factors that have been called forth, viral infections – particularly enteroviruses - have received overarching attention. While epidemiological studies show a temporal correlation between enteroviral infections and appearance of anti-islet auto-antibodies (aAbs) [5], investigations using the NOD mouse model suggest that enteroviral infections may accelerate rather than initiate T1D progression, as they are effective only once autoimmune T cells have already accumulated in the islets [6]. The pathophysiological mechanisms through which enteroviral infections may favor T1D development include promoting local islet inflammation, cytolytic effects on β cells and molecular mimicry [7]. This latter concept has been proposed based on aminoacid sequence homologies and/or immune cross-reactivity between viral and β-cell epitopes [8].

The role of bacterial infections as T1D triggers or accelerators have received comparatively less attention. *Mycobacterium avium* subspecies *paratuberculosis* (MAP) is the causative agent of paratuberculosis (Johne's disease), a chronic enteritis that affects dairy herds [9]. Environmental contamination with MAP is widespread, as MAP is detected in cattle's feces, soil, water (where it survives chlorination), it is shed into milk [10] and is found in commercially pasteurized dairy preparations [11] and meat products [12]. Although transmitted to man, MAP infection is asymptomatic in human carriers and is not therefore regarded as a zoonosis, nor subjected to eradication in contaminated animals.

Counting ∼1.8 million inhabitants, ∼3.5 millions sheeps and approximately two hundred thousand cattle, MAP exposure may be particularly high in the Western Mediterranean island of Sardinia, where it is estimated that ∼60% of flocks may be contaminated. Sardinia is also one of the regions with the highest incidence of T1D and multiple sclerosis (MS) worldwide, a notable exception in the north-south gradient followed by these autoimmune diseases.

Although evidence for a cause-effect relationship is lacking, MAP transmission to humans has long been associated with Crohn's disease both in Sardinia [13] and elsewhere [14]. We have recently proposed that MAP infection may be a potential candidate environmental trigger also for T1D. Our hypothesis is based on two key findings. First, MAP infection is highly prevalent in Sardinian T1D patients. Indeed, MAP DNA can be isolated from blood in 63% of Sardinian T1D patients, but only in 16% of healthy controls [15]; the MAP envelope protein MptD can be detected in the blood of 47.3% Sardinian T1D patients, but in a smaller proportion of type 2 diabetes (T2D) patients (7.7%) and healthy controls (12.6%) [16]; and MAP bacilli can be cultured from blood [16]. Second, this MAP infection triggers a specific humoral response, as Sardinian T1D patients display high frequencies of antibodies (Abs) against mycobacterial proteins (heparin-binding hemagglutinin, glycosyl transferase) [17], whole MAP lysates (70% Ab+ T1D patients vs 7.6% Ab+ healthy controls) [16] and the MAP-specific proteins MptD (MAP3733c) and MAP3738c when compared to T2D and healthy controls [16].

More recently, we have also documented an association between MAP DNA detected in blood and MS in Sardinian patients [18]. Thirty-two percent of MS patients, but only 2% of healthy controls, also harbored Abs recognizing the MAP2694 protein, which displays a 33% homology with the C region of the T-cell receptor (TCR) γ chain expressed by γδ T cells. The γδ T-cell subset patrols gut epithelial barriers and has been called into question in clearing infectious pathogens, most notably mycobacteria [19]. As depletion of γδ T cells in murine models of experimental autoimmune encephalomyelitis leads to disease development or exacerbation [20], we proposed that anti-MAP2694 Abs may cross-react with the γδ TCR, possibly leading to γδ T-cell depletion triggering increased MS susceptibility [18].

Another MAP protein, namely MAP3865c, displays a sequence homology with the β-cell antigen zinc transporter 8 (ZnT8), which is targeted by aAbs in T1D patients [21]. In light of this homology, we here sought to determine whether Abs recognizing MAP3865c are cross-reactive with ZnT8 epitopes. We show that Sardinian T1D patients specifically mount anti-MAP3865c Ab responses. Two immunodominant Ab epitopes were identified within the MAP3865c sequence and shown to be cross-reactive with the homologous ZnT8 sequences, raising the possibility of a molecular mimicry between mycobacterial and β-cell epitopes.

## Results

### Anti-MAP3865c Abs are highly prevalent in Sardinian T1D patients, but not in T2D patients

**Figure 1:**
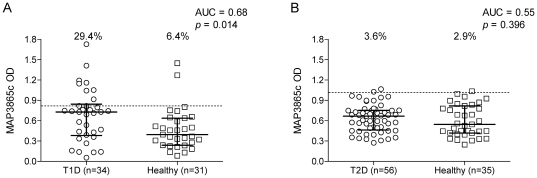
Prevalence of anti-MAP3865c Abs in Sardinian T1D and T2D patients. Sera were tested for their reactivity against plate-coated MAP3865c-MBP fusion protein. Ab distribution is shown for T1D (A) and T2D (B) patients compared to healthy controls. Dotted lines indicate the cut-off for positivity used in each assay, as calculated by ROC analysis. The percent fraction of Ab+ sera is indicated on top of each distribution, while bars indicate the corresponding median ± interquartile range. AUC and *p* values are given in the top right corner. Figures show representative experiments out of three performed.

The purified MAP3865c-MBP fusion protein was first used to screen by ELISA for the presence of serum anti-MAP3865c Abs. As shown in Fig. 1A, 29.4% of T1D patients displayed serum reactivity against MAP3865c compared to 6.4% of healthy controls (AUC 0.68, *p* = 0.014). This reactivity was specific of T1D patients, as it was not significantly different between T2D patients and controls (Fig. 1B; 3.6% vs 2.9%; AUC 0.55; *p* = 0.396).

**Figure 2:**
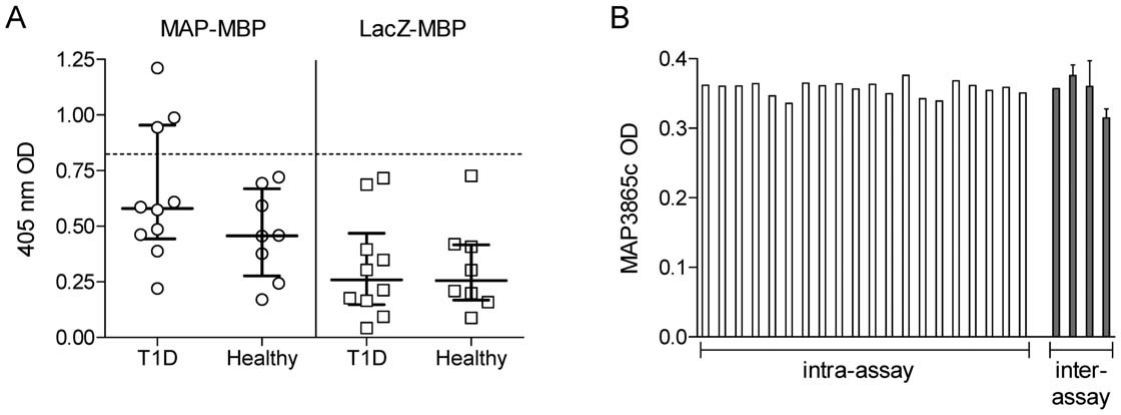
Validation of anti-MAP3865c Ab assays. (A) Reactivity against the MAP-MBP fusion protein is MAP-specific. Ab+ and Ab-negative sera from T1D and healthy donors were challenged either with the MAP-MBP fusion protein (as in Fig. 1) or with a LacZ-MBP control protein. The dotted line indicates the cutoff for positivity. (B) Intra- and inter-assay variability of MAP3865c ELISA Ab assays. For intra-assay variability (white bars), the same serum was tested in 20 replicate wells; bars show readouts of each single well. CV is 2.8%. For inter-assay variability (grey bars), the same serum was tested in 4 separate experiments; bars show mean ± SEM of triplicate wells from each experiment. CV is 7.4%.

Since the MAP3865c protein was fused with MBP, Ab+ and Ab-negative sera were tested against the LacZ-MBP control to exclude potential MBP-specific reactivities. A difference in Ab reactivity between Ab+ and Ab-negative sera and between T1D and healthy subjects was only observed when testing with the MAP-MBP protein, while the LacZ-MBP protein did not discriminate any positive sample using the same sera (Fig. 2A).

The ELISA assay employed displayed good reproducibility. For determination of intra-assay variability, a serum with MAP3865c Ab reactivity near the cut-off values was tested 20 times in a single experiment, giving a coefficient of variation (CV) of 2.8%. (Fig. 2B). The same serum tested in 4 separate experiments yielded an inter-assay CV of 7.4% (Fig. 2B).

As previously reported [15], the presence of MAP-specific IS900 DNA was also more prevalent among T1D patients (55.9%) than among T2D and healthy controls (7.0% and 20.0%, respectively; *p*<0.001). However, there was no correlation between positivity for anti-MAP3865c Abs and IS900 DNA (Table 1), although the frequency of Ab+ T1D patients was higher in the IS900 DNA+ group (7/34̧20.6% vs 3/34, 8.8%).

**Table 1:** Prevalence of MAP-specific IS900 DNA and of anti-MAP3865c Abs in the peripheral blood of T1D patients (n = 34).

	IS900 PCR+	IS900 PCR−
**MAP3865c Ab+**	20.6%	8.8%
**MAP3865c Ab−**	52.9%	17.7%

### Anti-MAP3865c Abs recognize an immunodominant transmembrane region homologous to ZnT8

**Figure 3:**
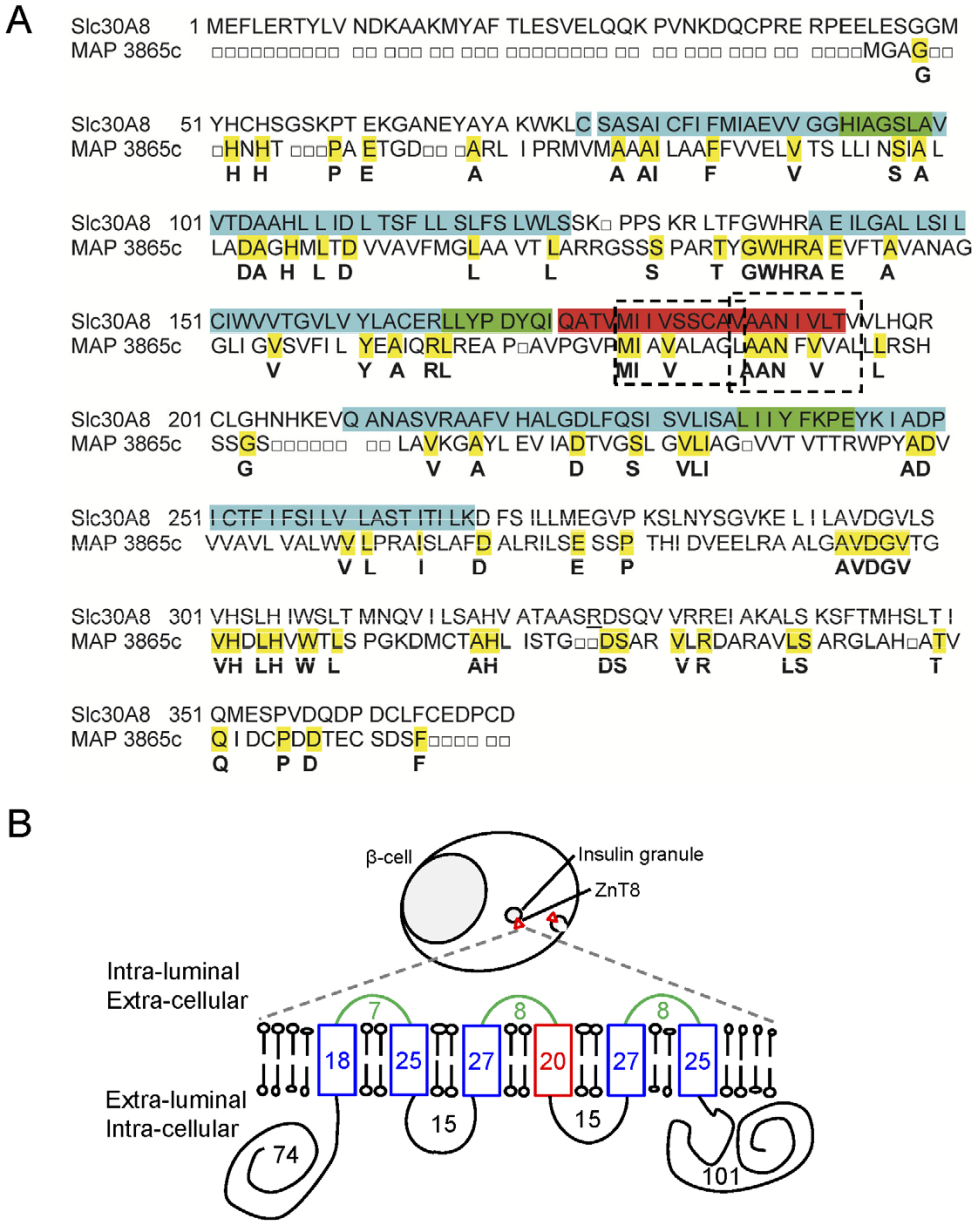
Homology between MAP3865c and ZnT8 proteins. (A) Aminoacid sequence alignment of ZnT8 (Slc30A8) and MAP3865c proteins. Conserved aminoacid residues are highlighted in yellow within the MAP3865c sequence and listed in bold below the two sequence alignment rows. The other color codes refer to the ZnT8 protein structure shown in (B): sequences highlighted in green belong to the 3 intra-luminal loops; the sequence in red belongs to the fourth transmembrane domain, while those in blue belong to the other transmembrane regions; sequences not highlighted fall within the 4 extra-luminal fragments. Dotted rectangles show the MAP3865c_125–133_/ZnT8_178–186_ and MAP3865c_133–141_/ZnT8_186–194_ peptides studied in subsequent experiments. The topology of the ZnT8 protein is also shown in panel (B), where the 3 intra-luminal loops (in green) become extracellularly exposed once the insulin granule is released. Conversely, the 4 extra-luminal domains (in black) are exposed to the cytosol and remain intracellular upon insulin exocytosis.

Scanning of the MAP3865c aminoacid sequence unraveled a 27.5% sequence identity with the human β-cell protein ZnT8 (Slc30A8) (Fig. 3A). To further explore the significance of this homology, we focused our analysis on one of the highly conserved regions (41.2% aminoacid identity) corresponding to the MAP3865c_125–141_ and ZnT8_178–194_ sequences. These sequences are located in one of the 6 membrane-spanning domains of the two proteins (Fig. 3B).

**Figure 4:**
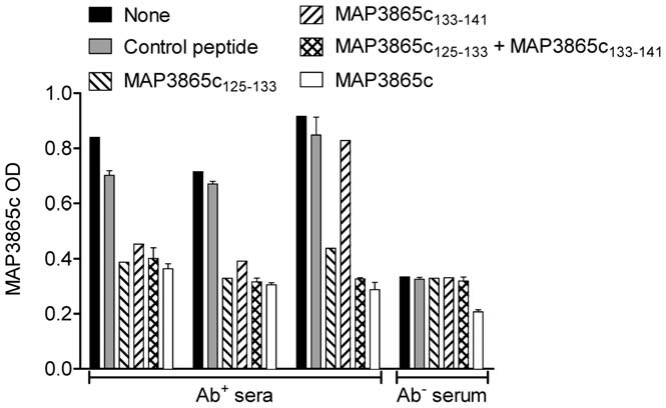
Ab reactivities against the MAP3865c protein are inhibited by MAP3865c_125–133_ and MAP3865c_133–141_ peptides. Ab+ and Ab-negative sera from T1D patients were pre-incubated overnight with saturating concentrations (5.5 µM) of MAP3865c_125–133_, MAP3865c_133–141_, the two peptides in combination, MAP3865c-MBP fusion protein and control or no peptide. Their reactivity on MAP3865c-MBP-coated ELISA plates was subsequently tested. Bars depict means ± SEM of triplicate wells and results are representative of three separate experiments.

Two nonamer peptides covering this region were synthesized: MAP3865c_125–133_ (MIAVALAGL) and MAP3865c_133–141_ (LAANFVVAL). Competition assays demonstrated that these epitopes are immunodominant Ab targets within the full-length MAP3865c protein, as sera pre-adsorbed with these peptides, either alone or in combination, were capable of blocking binding to the MAP3865c-MBP fusion protein, to a similar extent to what observed when pre-adsorbing sera with the MAP3865c-MBP protein itself (Fig. 4).

**Figure 5:**
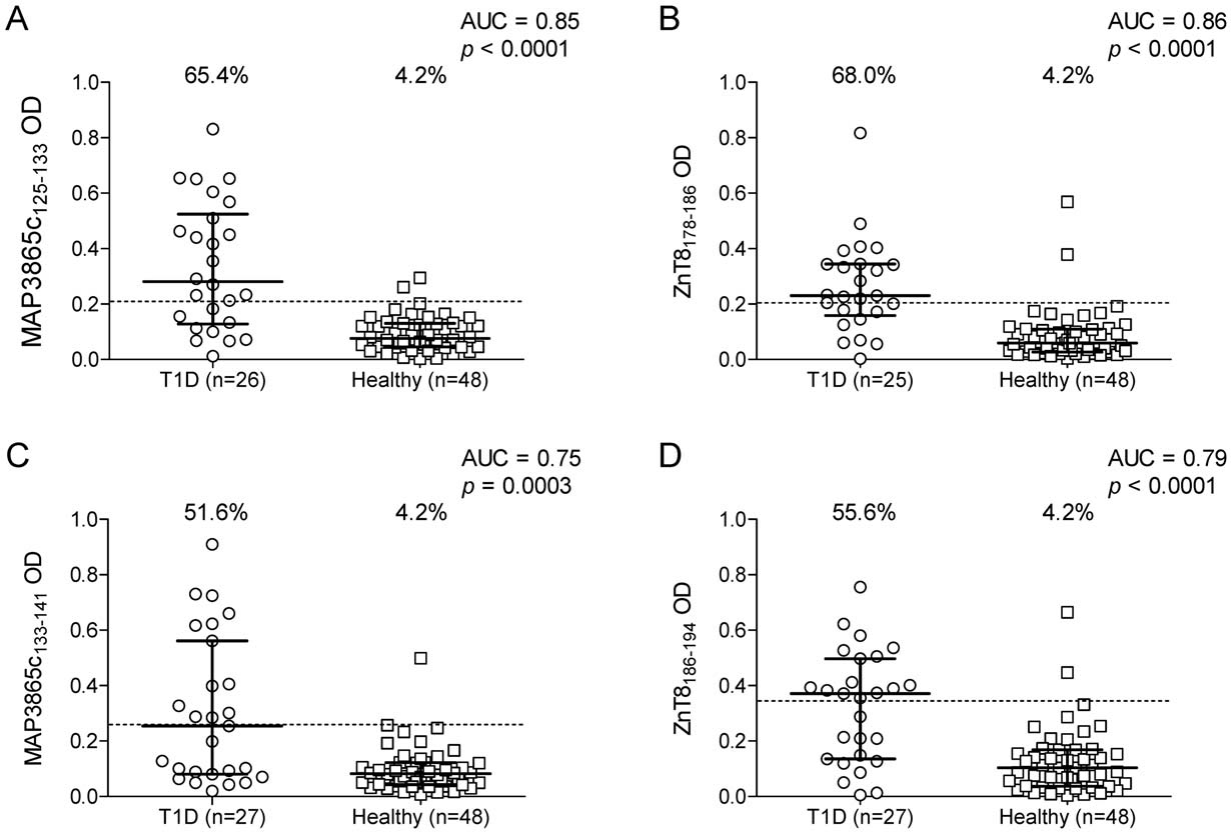
Prevalence of Abs against homologous MAP3865c and ZnT8 epitopes in T1D patients. Prevalence of Abs against MAP3865c_125–133_ (A) and its homologous ZnT8_178–186_ (B); and against MAP3865c_133–141_ (C) and its homologous ZnT8_186–194_ (D) in T1D and healthy subjects. Data representation is the same as in Fig. 1.

**Figure 6:**
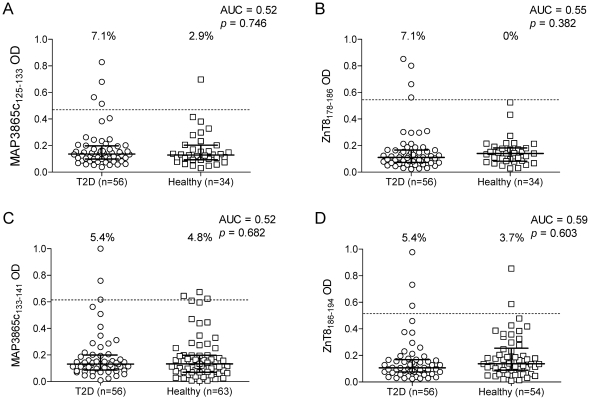
Prevalence of Abs against homologous MAP3865c and ZnT8 epitopes in T2D patients. Prevalence of Abs against MAP3865c_125–133_ (A) and its homologous ZnT8_178–186_ (B); and against MAP3865c_133–141_ (C) and its homologous ZnT8_186–194_ (D) in T2D and healthy subjects. Data representation is the same as in Fig. 1.

The homologous ZnT8 peptides corresponding to these MAP3865c sequences were further synthesized: ZnT8_178–186_ (MIIVSSCAV) and ZnT8_186–194_ (VAANIVLTV). Serum Ab reactivity against these four MAP3865c and ZnT8 peptides was further tested using the same ELISA assay. Also in this case, a significant difference in the frequency of Ab+ sera was observed between T1D and healthy subjects (Fig. 5). The homologous MAP3865c_125–133_ and ZnT8_178–186_ peptides (Fig. 5A–B) were recognized by 65.4% and 68.0% of T1D patients, but only in 4.2% of healthy controls (AUC 0.85 and 0.86, respectively; *p*<0.0001 for both). This serum Ab reactivity was also observed for the MAP3865c_133–141_ and ZnT8_186–194_ peptides (Fig. 5C–D), as 51.6% and 55.6% of T1D patients were Ab+, respectively, compared to 4.2% of healthy controls (AUC 0.75 and 0.79; *p* = 0.0003 and *p*<0.0001, respectively). As observed for the whole MAP3865c protein, this reactivity was specific of T1D patients, as it was not observed among T2D subjects (Fig. 6).

Comparison of Ab+ and Ab-negative T1D patients (Table 2) showed that anti-MAP3865c Ab+ patients had a significantly shorter disease duration than Ab-negative pairs (10.9±7.7 vs 17.9±10.0; *p* = 0.025). Similar trends were observed when comparing T1D patients harboring or not Abs against MAP3865c_125–133_ (12.6±8.8 vs 17.2±10.0; *p* = 0.068) and its homologous ZnT8_178–186_ (12.6±8.8 vs 18.0±10.0; *p* = 0.068), but not for Abs against MAP3865c_133–141_ (13.2±8.6 vs 17.5±10.4; *p* = 0.170) or its homologous ZnT8_186–194_ (13.8±12.3 vs 17.5±9.5; *p* = 0.296). A trend towards an older age at T1D diagnosis was also observed in patients positive for Abs against MAP3865c (22.9±9.6 vs 16.3±10.0; *p* = 0.072).

**Table 2:** T1D duration and age at T1D diagnosis in Ab+ and Ab-negative T1D patients.

	T1D duration (yrs)	Age at T1D diagnosis (yrs)
**MAP3865c Ab+**	10.9±7.7	22.9±9.6
**MAP3865c Ab−**	17.9±10.0	16.3±10.0
***p***	0.025	0.072
**MAP3865c_125–133_ Ab+**	12.6±8.8	19.8±11.0
**MAP3865c_125–133_ Ab−**	17.2±10.0	17.1±9.8
***p***	0.068	0.340
**ZnT8_178–186_ Ab+**	12.6±8.8	19.8±11.0
**ZnT8_178–186_ Ab−**	18.0±10.0	16.7±9.8
***p***	0.068	0.340
**MAP3865c_133–141_ Ab+**	13.2±8.6	20.8±10.2
**MAP3865c_133–141_ Ab−**	17.5±10.4	16.6±10.5
***p***	0.170	0.229
**ZnT8_186–194_ Ab+**	13.8±12.3	20.1±14.3
**ZnT8_186–194_ Ab−**	17.5±9.5	16.6±9.3
***p***	0.296	0.347

T1D patients whose Ab reactivities are shown in Figures 1 and 5 were compared using the Mann-Whitney U test. Mean ± SD are shown.

### Anti-MAP3865c and anti-ZnT8 Abs recognizing homologous sequences are cross-reactive

**Figure 7:**
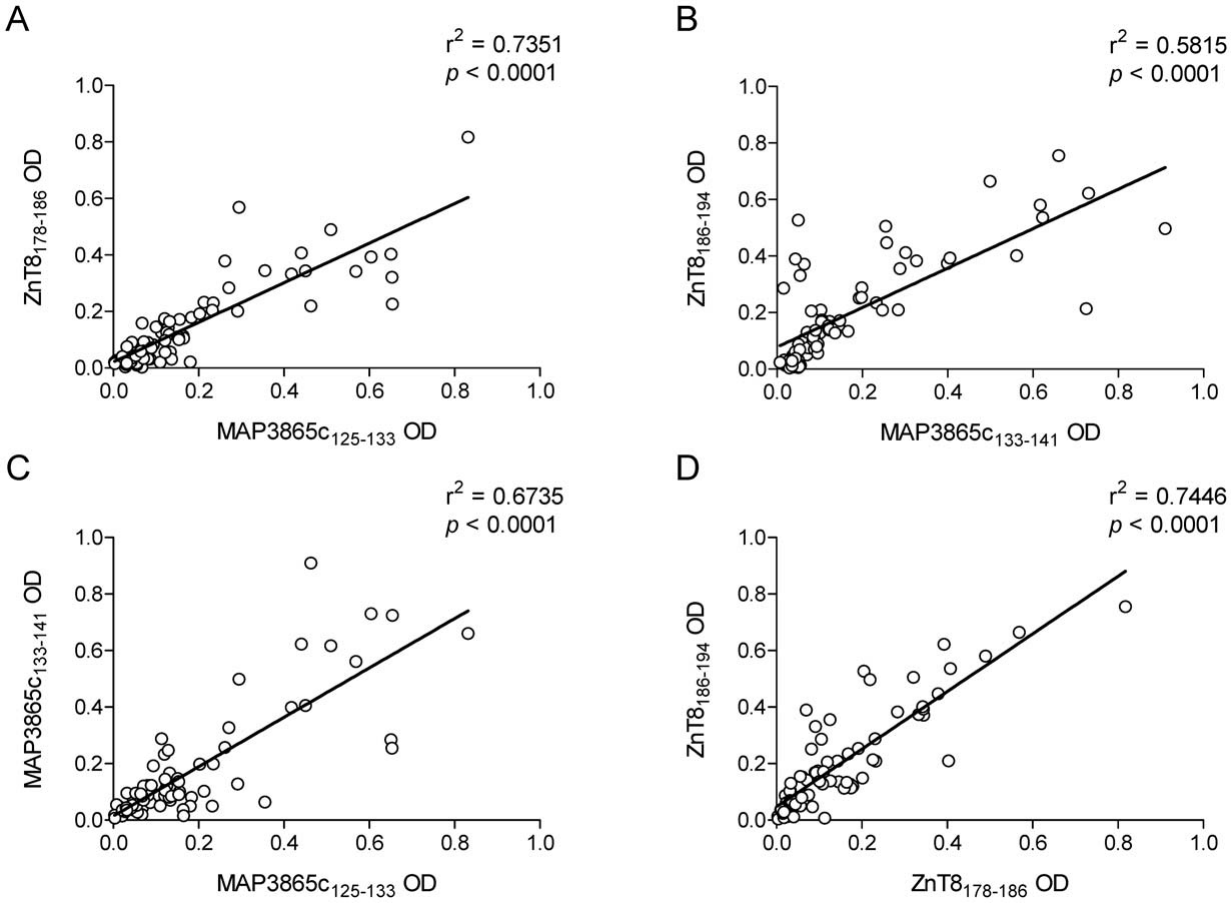
Correlation between titers of MAP3865c- and ZnT8-reactive Abs recognizing different epitopes. Correlations are shown between titers of Abs recognizing (A) MAP3865c_125–133_ and its homologous ZnT8_178–186_ epitope; (B) MAP3865c_133–141_ and its homologous ZnT8_186–194_ epitope; (C) MAP3865c_125–133_ and its consecutive MAP3865c_133–141_ epitope; (D) ZnT8_178–186_ and its consecutive ZnT8_186–194_ epitope. Each circle represents the titers of one T1D or healthy donor.

The similar frequencies of Abs recognizing MAP3865c and ZnT8 homologous regions among T1D patients (65.4–68.0% and 51.6–55.6%, respectively; Fig. 5) suggest that Abs targeting these epitopes could be cross-reactive. Indeed, there was a high degree of correlation between the titers of Abs recognizing MAP3865c and ZnT8 homologous sequences in both T1D patients and healthy controls (Fig. 7A–B; r^2^ = 0.74 for MAP3865c_125–133_ vs ZnT8_178–186_ and r^2^ = 0.58 for MAP3865c_133–141_ vs ZnT8_186–194_; *p*<0.0001). This correlation was maintained when the analysis was restricted to either T1D patients or healthy controls (data not shown). This demonstrates that anti-MAP3865c and anti-ZnT8 Abs recognizing homologous sequences segregate within the same sera. The same was true for Ab reactivities against the two neighboring regions MAP3865c_125–133_ and MAP3865c_133–141_ and for ZnT8_178–186_ and ZnT8_186–194_ (Fig. 7C–D; r^2^ = 0.67 and 0.74, respectively; *p*<0.0001), suggesting that recognition of these epitopes stems from an immune response against the whole MAP3865c/ZnT8 transmembrane region to which they belong.

**Figure 8:**
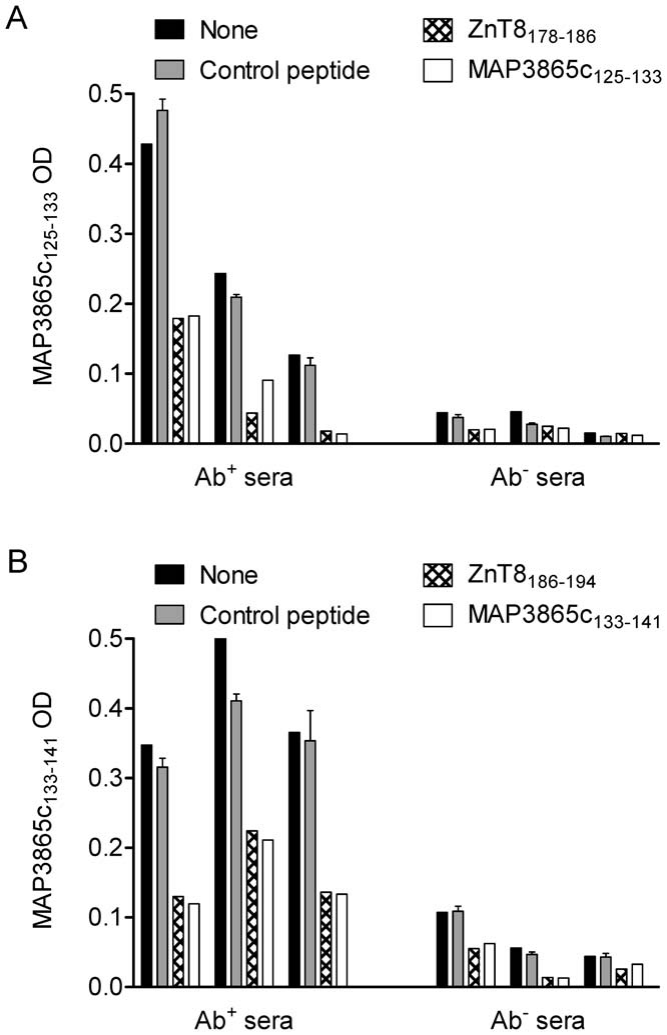
Ab reactivities against MAP3865c epitopes are inhibited by the homologous ZnT8 epitopes. (A) Ab+ and Ab-negative sera from T1D patients were pre-incubated overnight with saturating concentrations of MAP3865c_125–133_ (white bars), ZnT8_178–186_ (hatched bars), control (grey bars) or no peptide (black bars) and their reactivity on MAP3865c_125–133_-coated ELISA plates subsequently tested. (B) The same sera were preincubated with MAP3865c_133–141_ (white bars), ZnT8_186–194_ (hatched bars), control (grey bars) or no peptide (black bars) and their reactivity on MAP3865c_133–141_-coated ELISA plates subsequently tested. Bars depict means ± SEM of triplicate wells and results are representative of two separate experiments.

To verify whether co-segregation of these reactivities was due to Ab specificities cross-reacting between each other, competition experiments were performed. Anti-MAP3865c_125–133_-positive and -negative sera were pre-adsorbed overnight with different peptides, then subjected to ELISA on MAP3865c_125–133_-coated plates (Fig. 8A). While a control peptide did not cause any decrease in signal, both MAP3865c_125–133_ and its homologous ZnT8_178–186_ peptide strongly inhibited the MAP3865c_125–133_ reactivity to a similar extent (57–89%). The same observation was repeated with the MAP3865c_133–141_ reactivity, which was efficiently inhibited (55–66%) upon serum pre-adsorption with either MAP3865c_133–141_ or its homologous ZnT8_186–194_ (Fig. 8B). Taken together, these results demonstrate that anti-MAP and anti-ZnT8 Abs targeting homologous membrane-spanning sequences are cross-reactive.

## Discussion

Building up on our previous reports documenting a high prevalence of MAP infection and sero-reactivity in Sardinian T1D patients [15], [16], [17], [22], we here demonstrate that the MAP3865c protein is a target of Ab responses that cross-react with homologous ZnT8 sequences. MAP3865c is a 298 aminoacid 6-membrane-spanning channel which endows MAP with the ability to transport cations through the membrane, an important feature associated with intracellular survival of mycobacteria [23]. ZnT8 is a 369 aminoacid protein which belongs to the cation diffusion facilitator family of ZnT (Slc30) proteins. It displays a remarkably similar structure and function, allowing Zn^2+^ to accumulate in the insulin granules of pancreatic β cells. Zn^2+^ cations are essential to form hexavalent insulin storage crystals and, eventually, for effective insulin secretion [24]. Most of the 71 aminoacid difference in length between MAP3865c and ZnT8 is made up by the first extra-luminal domain, which is much shorter for MAP3865c (Fig. 3B).

To look for potential cross-reactive Ab epitopes, we focused our analysis on a trasmembrane region of high homology. Ab reactivities against peptide sequences of this region were even more prevalent in T1D patients than those against the whole MAP3865c protein, perhaps reflecting masking of these hydrophobic epitopes in the solubilized MAP3865c protein. Importantly, Abs against this membrane-spanning epitopes would not be detected by conventional anti-ZnT8 aAb assays, which employ a fusion protein combining the 4 extra-luminal domains of ZnT8 [21]. Other regions of high homology are mostly located in these extra-luminal domains, raising the possibility that other cross-reactive epitopes may be recognized by other Abs, including conventional anti-ZnT8 aAbs. Of further note, the transmembrane region identified here does not comprise the polymorphic ZnT8 R/W variant at position 325, which is located in the last extra-luminal domain, thus making it unlikely that the ZnT8 genetic background may shape these Ab reactivities, as described for conventional anti-ZnT8 aAbs [25].

The intestinal localization of MAP infection may also favor cross-reactivity with Abs and T cells recognizing ZnT8. Indeed, the first encounter with β-cell antigens takes place in pancreatic lymph nodes [26], which also drain intestinal tissues [27]. Epitope mimicry and spreading may be further favored by high precursor frequencies of ZnT8-reactive naïve T cells. As ZnT8 has not been found expressed by medullary thymic epithelial cells [28], negative selection of ZnT8-reactive T cells may be ineffective. Thus, tolerance to ZnT8 may heavily rely on peripheral mechanisms such as immune ignorance, which may be readily overcome by MAP infection. The intestinal localization of MAP infection may also give reason for the lack of correlation between MAP IS900 DNA and Ab detection. Not all MAP-infected individuals may mount systemic Ab responses detectable in blood, or they may develop Abs against other MAP antigens.

It is still premature to conclude whether MAP3865c-ZnT8 cross-reactivity implies an epitope mimicry phenomenon initiating or precipitating T1D. To this end, three key points remain to be addressed. First, MAP3865c-ZnT8 cross-reactivity was documented at the Ab level, but we did not yet explore potentially cross-reactive T-cell responses. CD4+ T-cell priming is an early event in the autoimmune cascade, providing help to B lymphocytes for differentiating into Ab-secreting plasma cells. Importantly, none of the ZnT8-derived CD4+ T-cell epitopes recently described [29] map to the Ab-targeted region here identified. Moreover, CD8+ T cells are additional key players in β-cell autoimmunity [30].

Second, is MAP infection and sero-reactivity already present before or at T1D onset, i.e. in at-risk and new-onset T1D subjects? Once T1D established, we cannot resolve whether MAP infection preceded or followed T1D onset, pointing either to a causal relationship with disease or to a consequence thereof. The observed correlation between anti-MAP Abs and shorter disease duration makes the first hypothesis more attractive. It may be argued that anti-ZnT8 aAbs typically appear later than insulin aAbs in at-risk children [21]. This could suggest that MAP infection generating cross-reactive anti-ZnT8 aAbs is a late event. However, this would rule out an initiating effect, but not a precipitating contribution to T1D development once autoimmunity has been initiated, as proposed for coxsackievirus infections [6], [7]. Moreover, the T1D patients studied here were adults, who are rarely insulin aAb+ and more frequently harbor anti-GAD and/or anti-IA-2 aAbs, which do not clearly preceed anti-ZnT8 [21]. Of further note, the relationship between the classical anti-ZnT8 aAbs and MAP cross-reactivity remains to be established, as the cross-reactive epitopes here identified are located outside the regions recognized by these aAbs. It is thus possible that early MAP infection may initially ignite immune responses against this transmembrane region, to only later spread to the extra-luminal epitopes covered by anti-ZnT8 aAb assays.

Third, it is well established that T cells are the key pathogenic effectors of T1D, while aAbs only play an accessory role which is matter of debate [31]. Therefore, to definitely prove a causal relationship beyond the correlation between MAP infection and T1D, mouse studies should be performed to document that MAP infection, MAP3865c immunization and/or MAP-reactive T cells induce disease. These experiments are difficult to perform for a number of reasons. The presence of ZnT8-specific aAbs or T cells has not yet been reported in NOD mice. Moreover, the Ab epitopes here identified are poorly conserved in the mouse. Of further note, we cannot discount the possibility of a direct cytotoxic effect of anti-MAP3865c/ZnT8 cross-reactive Abs through binding to β cells. The transmembrane location of these epitopes may allow Abs to exert an agonistic or antagonistic effect on Zn^2+^ transport through the ZnT8 channel, possibly impinging on the β-cell secretory capacity.

The majority – but not all [32] – of studies on Sardinian migrants suggests a predominant weight of genetic factors on T1D pathogenesis, as disease incidence in the hosting region remains similar to that of Sardinia [33]. This observation is at variance with most T1D migrant studies [3] and with comparisons of T1D incidence between neighboring regions of uniform genetic background and diverse environmental exposure [34], which instead point to a prominent role of environmental factors. The possible involvement of MAP in the pathogenesis of Sardinian T1D may reconcile this conundrum. As MAP has been shown to pass into human breast milk [35], children born outside Sardinia from Sardinian mothers could still be more exposed to MAP infection. Moreover, Sardinian migrants frequently maintain regular interactions with their homeland relatives, including exchange of regional dairy and meat products which may contribute in maintaining MAP exposure.

Another open question is whether our current observations are specific to the Sardinian population or can be replicated elsewhere. Although MAP exposure is particularly high in Sardinia, contamination of food products with MAP has been documented worldwide. While we are not aware of studies performed in other areas at very high T1D incidence such as Scandinavia, studies conducted in UK document a MAP prevalence in herds and dairy products similar to what found in lower incidence countries [12]. Thus, the environmental prevalence of MAP does not systematically parallel that of T1D, suggesting that other factors may be at play. As suggested for enteroviral infections [6], [7], timing of MAP infection may be one such factor, making epitope mimicry effective only once an inflammatory milieu is established, once β-cell autoimmunity is initiated and/or once a critical mass of ZnT8-reactive T cells have already accumulated. The chronic nature of MAP infection may also perpetuate availability of cross-reactive epitopes. Ultimately, the interaction between MAP environmental exposure and a susceptible genetic background such as the Sardinian one may be critical, as children whose parents have migrated to Sardinia do not acquire a higher T1D risk [36]. Indeed, the gene pool of Sardinians is highly distinct from that of all other Mediterranean regions, probably reflecting a genetic drift entrenched over centuries by geographical isolation, epidemic misfortunes and endogamy [37]. Genetic susceptibility may be conferred by polymorphic loci involved in clearance of intracellular bacteria, such as the *SLC11A1* locus (previously known as *NRAMP1*). *SLC11A1* polymorphisms have been associated with T1D in Sardinian [38] and Japanese [39] patients and in NOD mice [40], as well as with susceptibility to mycobacterial infections [41]. SLC11A1 is a transporter of divalent cations in the late endosomal/lysosomal compartments of phagocytes [42]. Transport of divalent cations contributes to enhanced phagosomal acidification [43], thus impairing survival of intracellular pathogens [42] and modifying processing and presentation of certain epitopes, including β-cell derived ones [43]. Another intriguing observation is that the long-standing diatribe as to whether early childhood exposure to cow's milk predisposes to islet Ab seroconversion and T1D has remained unsettled [44], [45]. MAP contamination of milk supplies and the interaction between MAP environmental exposure and a susceptible genetic ground may offer an alternative interpretation.

Finally, the individual genetic background over which MAP exposure builds up may dictate susceptibility to other autoimmune and inflammatory diseases such as MS and Crohn's disease, which have also been associated with MAP infection in Sardinia [13], [18] and elsewhere [14]. Further investigations outside Sardinia and comprehensive dissection of the groove between genetic and environmental factors will be critical in deciphering the significance of these findings in T1D pathogenesis.

## Materials and Methods

### Patient and control serum samples

T1D patients (n = 34; mean age 34.5±7.7 years, mean age at onset 17.5±10.2 years, mean T1D duration 16.8±9.9 years) and T2D patients (n = 56; mean age 64.8±8.6 years, mean age at onset 56.4±9.2 years, mean T2D duration 8.5±5.3 years) diagnosed according to the American Association of Diabetes criteria [46] and healthy blood donors (n = 63) age-matched with T1D patients (mean age 38.5±12.0 years; *p* = 0.102) were recruited at the University Hospital of Sassari. Written informed consents were obtained before blood drawing and the study was approved by the University Ethics Committee. Serum samples were processed as previously described [16].

### Construction of the pMAL-MAP3865c expression vector

MAP DNA was extracted with the detergent cetyltrimethylammonium bromide (Sigma). The full-length *MAP3865c* gene was amplified by PCR from the MAP DNA ATCC43015 with a sense primer (5′-GCGCGAATTCATGGGCGCCGGCCACAACCACAC-3′) and an antisense primer (5′- GCGCCTGCAGTCATCAGAAGCTGTCGGAGCACTC-3′), where underlined sequences are *EcoRI* and *PstI* restriction sites, respectively. The *MAP3865c* coding sequence was cloned into pMALc2X (New England Biolabs) next to a maltose-binding protein (MBP) sequence, and the ligation mix was used to transform *E. coli* K12 TB1 competent cells. Transformants were screened by plating the electroporated K12 TB1 cells on rich medium (10 g/l tryptone, 5 g/l yeast extract, 5 g/l NaCl) supplemented with ampicillin (100 µg/ml). The coding sequence of the cloned *MAP3865c* gene fully matched the published sequence of the *MAP3865c* gene of *M. paratuberculosis* K10 (GenBank accession number: NC002944).

### MAP3865c protein expression and purification

*E. coli* TB1 cells harboring the expression plasmid were grown at 37°C and a single colony was used to inoculate rich medium containing 1 g/l ampicillin and 2 g/l glucose. MAP3865c-MBP fusion protein expression was induced by addition of 0.3 mM isopropyl-β-D-thiogalactopyranoside (Sigma). After 2 h, cells were harvested, resuspended in 20 ml of column buffer (20 mM Tris-HCl, 200 mM NaCl, 1 mM ethylenediaminetetraacetic acid (EDTA), 1∶100 Sigma protease inhibitor cocktail) and frozen at −20°C. The following day, cells were lysed by sonication. Debris were removed by centrifugation, supernatants were diluted 1∶5 with column buffer, loaded on a column charged with amylose resin (New England Biolabs) and washed 5 times. The fusion protein was eluted with column buffer containing 10 mM maltose. The MAP3865c-MBP fusion protein migrated at the expected molecular mass of 72.5 kD in sodium dodecyl sulfate polyacrylamide gel electrophoresis.

### Peptides

Peptides MAP3865c_125–133_ (MIAVALAGL) and MAP3865c_133–141_ (LAANFVVAL) along with their respective homologous peptides ZnT8_178–186_ (MIIVSSCAV) and ZnT8_186–194_ (VAANIVLTV) were synthesized at >85% purity (GL Biochem). Conserved aminoacid residues are underlined. Stock solutions (10 mM in dimethyl sulfoxide) were stored in single-use aliquots at −80°C.

### Enzymatic immunoassay

An indirect enzyme-linked immunosorbent assay (ELISA) were set up to detect Abs specific for MAP3865c protein and peptides. Ninety-six-well Nunc immunoplates were coated overnight at 4°C with 5 µg/ml of recombinant MAP3865c-MBP fusion protein or 10 µg/ml of peptides diluted in 0.05 M carbonate–bicarbonate buffer, pH 9.5 (Sigma). Plates were then blocked for 1 h at room temperature with 5% non-fat dried milk (Sigma) and washed twice with phosphate-buffered saline (PBS) containing 0.05% Tween-20 (PBS-T). Serum samples were subsequently added at 1∶100 dilution in PBS-T for 2 h at room temperature. After 5 washes in PBS-T, 100 µl of alkaline phosphatase-conjugated goat anti-human immunoglobulin G polyclonal Ab (1∶1000; Sigma) was added for 1 h at room temperature. Plates were washed again 5 times in PBS-T and para nitrophenylphosphate (Sigma) added as substrate for alkaline phosphatase. Plates were incubated at 37°C in the dark for 3–6 min and the absorbance at 405 nm read on a VERSATunable Max microplate reader (Molecular Devices). Negative control wells were obtained by incubation of immobilized protein or peptides with secondary Ab alone, and their mean values subtracted from all samples. Positive control sera were also included in all experiments. Results are expressed as means of triplicate 405 nm optical density (OD) values.

### Competition assays

Competition assays were performed by pre-incubating sera overnight at 4°C with saturating concentrations (5–20 µM, titrated for each individual serum) of MAP peptides, the corresponding ZnT8 peptides, irrelevant peptide (MAP3865c_211–217_, ILSESSP), no peptide, or MAP3865c-MBP fusion protein, as previously described [47]. Sera were then subjected to ELISA on plates coated with MAP3865c-MBP, MAP3865c_125–133_ or MAP3865c_133–141_, as above.

### MAP IS900 PCR

The presence of MAP-specific DNA in blood samples was detected by PCR amplification of IS900 sequences, as previously described [13].

### Statistical analyses

Receiver operator characteristic (ROC) curves were used to score the performance of each single ELISA in discriminating T1D or T2D patients from healthy controls. AUC was calculated assuming a non-parametric distribution of results. Thus, an AUC of 1.0 would indicate that the assay achieved 100% accuracy in identifying patients; an AUC of 0.5 would indicate that the assay gave no difference between patients and controls; and an AUC of 0 would indicate that the assay gave a positive result for controls and a negative result for patients. The cut-off for positivity in each assay was set at ≥93% specificity (i.e. Ab+ healthy controls ≤7%) and the corresponding sensitivity (i.e. percent of Ab+ patients) calculated accordingly. Clinical characteristics of Ab+ and Ab-negative patients were compared using the Mann-Whitney U test.
